# Specific mortality in patients with diffuse large B-cell lymphoma: a retrospective analysis based on the surveillance, epidemiology, and end results database

**DOI:** 10.1186/s40001-024-01833-4

**Published:** 2024-04-20

**Authors:** Hui Xu, Rong Yan, Chunmei Ye, Jun Li, Guo Ji

**Affiliations:** 1 Department of Hematology, Taixing People’s Hospital, No. 98, Runtai South Road, Taixing, 225400 Jiangsu China; 2 Taixing People’s Hospital, Taixing, Jiangsu China

**Keywords:** Diffuse large B-cell lymphoma, Competing risk model, Fine and Gray regression, SEER database

## Abstract

**Background:**

The full potential of competing risk modeling approaches in the context of diffuse large B-cell lymphoma (DLBCL) patients has yet to be fully harnessed. This study aims to address this gap by developing a sophisticated competing risk model specifically designed to predict specific mortality in DLBCL patients.

**Methods:**

We extracted DLBCL patients’ data from the SEER (Surveillance, Epidemiology, and End Results) database. To identify relevant variables, we conducted a two-step screening process using univariate and multivariate Fine and Gray regression analyses. Subsequently, a nomogram was constructed based on the results. The model’s consistency index (C-index) was calculated to assess its performance. Additionally, calibration curves and receiver operator characteristic (ROC) curves were generated to validate the model’s effectiveness.

**Results:**

This study enrolled a total of 24,402 patients. The feature selection analysis identified 13 variables that were statistically significant and therefore included in the model. The model validation results demonstrated that the area under the receiver operating characteristic (ROC) curve (AUC) for predicting 6-month, 1-year, and 3-year DLBCL-specific mortality was 0.748, 0.718, and 0.698, respectively, in the training cohort. In the validation cohort, the AUC values were 0.747, 0.721, and 0.697. The calibration curves indicated good consistency between the training and validation cohorts.

**Conclusion:**

The most significant predictor of DLBCL-specific mortality is the age of the patient, followed by the Ann Arbor stage and the administration of chemotherapy. This predictive model has the potential to facilitate the identification of high-risk DLBCL patients by clinicians, ultimately leading to improved prognosis.

**Supplementary Information:**

The online version contains supplementary material available at 10.1186/s40001-024-01833-4.

## Introduction

DLBCL, known as diffuse large B-cell lymphoma, is a highly heterogeneous disease and is the most common type of non-Hodgkin’s lymphoma, accounting for approximately 30–40% of all lymphoma cases [1]. While there have been significant advancements in the diagnosis and treatment of DLBCL in recent years, it is disheartening to note that 40–50% of patients with DLBCL still remain incurable [2]. For patients who experience a relapse or have refractory DLBCL, the prognosis is generally poor [3]. Hence, it becomes imperative to identify highly specific and sensitive prognostic markers that can effectively identify high-risk patients, thereby enabling improved treatment decisions and ultimately enhancing patient survival.

Several studies have examined prognostic factors in patients with DLBCL [2, 4–7]. However, many of these studies have relied on the conventional Cox proportional hazards model [7, 8]. It is important to note that competing mortality events frequently arise during the analysis of survival data. Yet, the traditional Cox regression often fails to consider the occurrence of these competing mortality events, leading to potential misjudgment of patient prognosis, irrespective of the independence between such events. If a patient dies from causes other than DLBCL, and the Cox regression fails to account for these competing mortality events, it introduces bias into the analysis results. The Fine and Gray model enables us to analyze data while taking into account competing risks. Similar to the Cox model, the Fine and Gray model utilizes a risk set function, but it also incorporates the concept of competition between different types of events. This model estimates the probability of each event by comparing the event-specific risk set function with the overall risk set function, while accounting for the impact of other event types. Competing risk models, specifically the Fine and Gray proportional hazards model, demonstrate excellent capability in addressing the correlation between cancer outcomes and competing events, ultimately leading to a remarkable enhancement in the accuracy of prognostic analysis [9]. Despite its potential, this methodology remains largely underutilized. Leveraging the SEER database, a comprehensive and extensive multi-center database with credible data sources, this study aims to establish a competing risk model based on DLBCL patients. The objective is to investigate the factors that influence cause-specific mortality in DLBCL patients.

## Methods

### Study cohort

We extracted data from the SEER database [Incidence—SEER Research Plus Data, 17 Registries, Nov 2021 Sub (2000–2019)] using SEER Stat (Version 8.4.1) software. The data pertain to patients diagnosed with DLBCL between 2000 and 2015. To ensure data quality, patients with less than 1 month of follow-up and those with one or more missing variables were excluded from the analysis. The collected data encompassed demographic information such as sex, race, age, marital status, median household income, and place of residence. It also included tumor characteristics such as site, primary site, presence of B symptoms, number of malignant tumors, and whether it was the first primary tumor. Additionally, the data recorded the Ann Arbor Stage, surgical and chemoradiotherapy information including surgery, radiation, chemotherapy, the sequence of systemic therapy and surgery, and treatment timing. Furthermore, the cause of death and follow-up information were documented. The diagnosis of diffuse large B-cell lymphoma (DLBCL) was made based on the criteria outlined in the International Classification of Diseases for Oncology, 3rd Edition (ICD-O-3). The staging of lymphoma was determined using the Ann Arbor stage system (AASS). Regarding the analysis of continuous variables, the subjects were categorized into different groups based on their treatment timing (the interval between the diagnosis and initiation of treatment): more than 1 month and 1 month or less. The subjects were also divided into 5 age groups: 0–19 years, 20–39 years, 40–59 years, 60–79 years, and 80–100 years. Furthermore, based on the median annual household income, the subjects were divided into 3 groups: less than $50,000, $50,000–$74,999, and greater than $75,000.

### Statistical analysis

The study cohort was divided into a training cohort and a validation cohort in a ratio of 7:3. The purpose of this division was to use the training cohort to train the model and the validation cohort to test the model. All patient features were divided into the training and validation cohorts, and the balance of the data was assessed by comparing the differences between the two groups. Categorical variables were presented as frequencies and percentages (25%), and chi-square tests were used to compare the differences between the two groups. Normally distributed continuous variables were displayed as means and standard deviations [Mean (S.E.)], and *t*-tests were used to compare the differences. Non-normally distributed continuous variables were presented as medians and quartiles (median [IQR]), and rank sum tests were used to compare the differences. In the competing risk model, the outcome event of interest was death from DLBCL, and death from other causes was treated as a competing event. Variables were screened in two steps using univariate and multivariate Fine and Gray regression analyses. Variables that were statistically significant in the univariate analysis were included in the multivariate analysis. The variables that remained statistically significant in the multivariate analysis were used to construct a competing risk model and develop a corresponding nomogram. The model’s C-index was calculated, and its predictions were compared with the observed actual values. Calibration curves and ROC curves were plotted to assess the consistency and accuracy of the model. All statistical analyses were performed using R 4.2.1 (https://www.r-project.org/). The Fine and Gray regression analysis and competing risk modeling were conducted using the riskRegression (2021.10.10) software package. The pmsampsize (1.1.3) package was used to calculate the sample size and plot ROC and calibration curves, while the rms package was used for nomogram plotting.

## Results

### Patient features

**Fig. 1:**
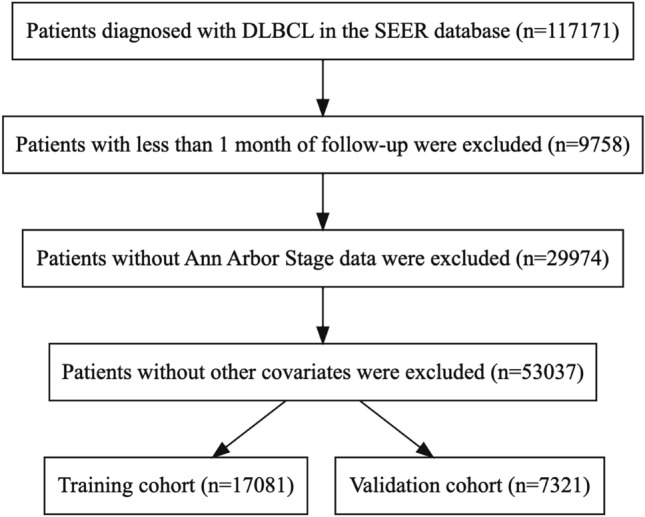
Patient selection flowchart

A total of 117,171 patients diagnosed with diffuse large B-cell lymphoma (DLBCL) were identified from the dataset titled “Incidence—SEER Research Plus Data, 17 Registries, Nov 2021 Sub (2000–2019)” that was submitted to the SEER database in 2021. Patients who had less than 1 month of follow-up (*N* = 9758), patients without Ann Arbor Stage data (*N* = 29,974), and patients with one or more missing variables (*N* = 53,037) were excluded from the study (Fig. 1). Eventually, a total of 24,402 patients were included in this study. Among them, 6459 died from DLBCL and 4076 died from other causes. The median survival time for patients in the entire study cohort was 58 months (IQR: [16.00, 83.00]). The majority of patients were between the ages of 60 and 79 years (48.0%), and there was a higher proportion of men compared to women (57.1%). The characteristics of patients in both the training cohort and the validation cohort are described in Table 1. There were no statistically significant differences in each variable between the two cohorts (*P* > 0.05), indicating a balanced distribution of data.

**Table 1:** Description of features of all patients

Factors	Overall	Training cohort	Validation cohort	*P* value
*N*	24,402	17,081	7321	
Time	58 [16, 83]	58 [16, 83]	58 [17, 83]	0.803
Age				
0–19 years	308 (1.3%)	211 (1.2%)	97 (1.3%)	0.781
20–39 years	1972 (8.1%)	1389 (8.1%)	583 (8.0%)	
40–59 years	6450 (26%)	4490 (26%)	1960 (27%)	
60–79 years	11,702 (48%)	8227 (48%)	3475 (47%)	
80–100 years	3970 (16%)	2764 (16%)	1206 (16%)	
Ann Arbor stage				
Stage I	5418 (22%)	3772 (22%)	1646 (22%)	0.908
Stage II	5372 (22%)	3769 (22%)	1603 (22%)	
Stage III	5016 (21%)	3522 (21%)	1494 (20%)	
Stage IV	8596 (35%)	6018 (35%)	2578 (35%)	
B symptoms	7959 (33%)	5547 (32%)	2412 (33%)	0.5
Chemotherapy				
With	22,728 (93%)	15,909 (93%)	6819 (93%)	1
Without	1674 (6.9%)	1172 (6.9%)	502 (6.9%)	
First primary tumor	19,747 (81%)	13,807 (81%)	5940 (81%)	0.6
Marital status				
Divorced	2023 (8.3%)	1384 (8.1%)	639 (8.7%)	0.381
Married	13,727 (56%)	9577 (56%)	4150 (57%)	
Other	1151 (4.7%)	810 (4.7%)	341 (4.7%)	
Separated	226 (0.9%)	163 (1.0%)	63 (0.9%)	
Single	4324 (18%)	3061 (18%)	1263 (17%)	
Widowed	2951 (12%)	2086 (12%)	865 (12%)	
Median household income				
< $50,000	3401 (14%)	2364 (14%)	1037 (14%)	0.767
> $75,000	7138 (29%)	5011 (29%)	2127 (29%)	
$50,000–$74,999	13,863 (57%)	9706 (57%)	4157 (57%)	
Number of malignancies				
> 1	6792 (28%)	4775 (28%)	2017 (28%)	0.529
1	17,610 (72%)	12,306 (72%)	5304 (72%)	
Place of residence				
Metropolitan	21,659 (89%)	15,168 (89%)	6491 (89%)	0.772
Nonmetropolitan	2743 (11%)	1913 (11%)	830 (11%)	
Primary site				
Digestive system	978 (4.0%)	684 (4.0%)	294 (4.0%)	0.166
Intra-abdominal lymph nodes	1557 (6.4%)	1113 (6.5%)	444 (6.1%)	
Intrathoracic lymph nodes	655 (2.7%)	460 (2.7%)	195 (2.7%)	
Lymph nodes of axilla or arm	455 (1.9%)	321 (1.9%)	134 (1.8%)	
Lymph nodes of head, face and neck	1634 (6.7%)	1120 (6.6%)	514 (7.0%)	
Lymph nodes of inguinal region or leg	574 (2.4%)	380 (2.2%)	194 (2.6%)	
Lymph nodes of multiple regions	8045 (33%)	5688 (33%)	2357 (32%)	
Nervous system	6 (< 0.1%)	4 (< 0.1%)	2 (< 0.1%)	
Other	10,318 (42%)	7175 (42%)	3143 (43%)	
Pelvic lymph nodes	180 (0.7%)	136 (0.8%)	44 (0.6%)	
Race				
Asian	2087 (8.6%)	1464 (8.6%)	623 (8.5%)	0.139
Black	1744 (7.1%)	1260 (7.4%)	484 (6.6%)	
Other	224 (0.9%)	150 (0.9%)	74 (1.0%)	
White	20,347 (83%)	14,207 (83%)	6140 (84%)	
Radiation				
With	5204 (21%)	3665 (21%)	1539 (21%)	0.458
Without	19,198 (79%)	13,416 (79%)	5782 (79%)	
Sex				
Female	10,480 (43%)	7305 (43%)	3175 (43%)	0.392
Male	13,922 (57%)	9776 (57%)	4146 (57%)	
Site				
Extra nodal	7050 (29%)	4910 (29%)	2140 (29%)	0.452
Nodal	17,352 (71%)	12,171 (71%)	5181 (71%)	
Surgery				
With	5252 (22%)	3657 (21%)	1595 (22%)	0.523
Without	19,150 (78%)	13,424 (79%)	5726 (78%)	
The sequence of systemic therapy and surgery				
No systemic therapy and/or surgery	19,384 (79%)	13,582 (80%)	5802 (79%)	0.878
Other	27 (0.1%)	18 (0.1%)	9 (0.1%)	
Systemic therapy after surgery	4812 (20%)	3354 (20%)	1458 (20%)	
Systemic therapy before surgery	122 (0.5%)	89 (0.5%)	33 (0.5%)	
Systemic therapy both before and after surgery	57 (0.2%)	38 (0.2%)	19 (0.3%)	
Treatment timing				
$$\le$$ 1 month	11,114 (46%)	7803 (46%)	3311 (45%)	0.521
> 1 month	13,288 (54%)	9278 (54%)	4010 (55%)	
Status				
0	13,867 (57%)	9707 (57%)	4160 (57%)	1
1	6459 (26%)	4521 (26%)	1938 (26%)	
2	4076 (17%)	2853 (17%)	1223 (17%)	

### Feature selection

The selection of features was carried out using univariate and multivariate Fine and Gray regression analyses. In the univariate analyses, only variables that showed statistical significance were included in the multivariate analyses. Similarly, in the multivariate analyses, only variables that showed statistical significance were included in the final model. The univariate analysis revealed that 14 variables were found to be statistically significant and were, therefore, considered potential risk factors for cause-specific mortality in DLBCL patients. These variables included race, tumor site (extranodal or nodal), primary site, Ann Arbor stage, whether surgery was performed, whether radiation therapy was administered, whether chemotherapy was administered, sequence of systemic therapy and surgery, treatment timing, presence of B symptoms, whether it was the first primary tumor, age, marital status, and median annual household income. Upon conducting a multivariate analysis with the above variables included in the adjusted model, it was revealed that 13 variables remained statistically significant and were identified as independent risk factors for cause-specific mortality in DLBCL patients (Additional file 1: Table S1). These variables included race, tumor site (extranodal or nodal), Ann Arbor stage, whether surgery was performed, whether radiation therapy was administered, whether chemotherapy was administered, sequence of systemic therapy and surgery, treatment timing, presence of B symptoms, whether it was the first primary tumor, age, marital status, and median annual household income. These 13 variables were further included in the competing risk model (Table 2). Furthermore, to effectively compare the disparities between Fine and Gray regression and Cox regression, we incorporated the aforementioned variables into the multivariate Cox regression analysis. The findings revealed that age, Ann Arbor Stage, b symptoms, absence of chemotherapy, absence of radiation, absence of surgery, the sequence of systemic therapy and surgery, and treatment timing exerted a more prominent influence on the risk of all-cause mortality (in the Cox proportional risk model) compared to the risk of DLBCL-specific mortality (in the Competing Risk Model) (refer to Table 3).

**Table 2:** The competing risk model

Factors	Levels	Coefficient	HR (95% CI)	Se (coefficient)	*Z*	*P* value
Age	0–20 years (reference)					
	20–39 years	0.3316	1.3931	0.258	1.2849	0.2000
	40–59 years	0.8204	2.2715	0.249	3.2946	0.0010
	60–79 years	1.3706	3.9379	0.2484	5.5176	< 0.0001
	80–100 years	1.9028	6.7044	0.2504	7.5998	< 0.0001
Ann Arbor stage	Stage I (reference)					
	Stage II	0.363	1.4376	0.0553	6.5694	< 0.0001
	Stage III	0.5625	1.7551	0.0568	9.9062	< 0.0001
	Stage IV	0.8849	2.4227	0.0511	17.3085	< 0.0001
B symptoms	No (reference)					
	Yes	0.2803	1.3235	0.0319	8.7865	< 0.0001
Chemotherapy	With (reference)					
	Without	0.6221	1.8628	0.0646	9.6246	< 0.0001
First primary tumor	No (reference)					
	Yes	− 0.1318	0.8765	0.0365	− 3.6142	0.0003
Marital status	Divorce (reference)					
	Married	− 0.1438	0.866	0.0537	− 2.68	0.0074
	Other	− 0.2752	0.7595	0.0901	− 3.0547	0.0023
	Separated	− 0.041	0.9598	0.1691	− 0.2426	0.8100
	Single	− 0.1127	0.8934	0.066	− 1.7078	0.0880
	Widowed	− 0.0435	0.9574	0.0641	− 0.6796	0.5000
Median household income	< $50,000 (reference)					
	> $75,000	− 0.2132	0.808	0.0494	− 4.3183	< 0.0001
	$50,000–$74,999	− 0.0951	0.9093	0.0441	− 2.1563	0.0310
Race	Asian (reference)					
	Black	− 0.0613	0.9405	0.0771	− 0.7954	0.4300
	Other	− 0.5926	0.5529	0.2064	− 2.8707	0.0041
	White	− 0.2131	0.8081	0.0528	− 4.0324	0.0001
Radiation	With (reference)					
	Without	0.1675	1.1824	0.0414	4.0418	0.0001
Site	Extranodal (reference)					
	Nodal	0.1227	1.1305	0.0374	3.2766	0.0011
Surgery	With (reference)					
	Without	0.3059	1.3578	0.0724	4.2218	< 0.0001
The sequence of systemic therapy and surgery	No systemic therapy and/or surgery (reference)					
	Other	0.777	2.175	0.3673	2.1155	0.0340
	Systemic therapy after surgery	− 0.0027	0.9973	0.0709	− 0.0387	0.9700
	Systemic therapy before surgery	0.3343	1.3969	0.2259	1.4799	0.1400
	Systemic therapy both before and after surgery	0.2455	1.2783	0.2919	0.8412	0.4000
Treatment timing	< 1 month (reference)					
	> 1 month	− 0.2386	0.7877	0.0329	− 7.2582	< 0.0001

**Table 3:** Comparison of multivariate competing risk analysis and multivariate Cox regression analysis

Factors	Levels	Competing risk model		Cox proportional risk model	
		HR (95% CI)	*P* value	HR (95% CI)	*P* value
Age	0–20 years (reference)				
	20–39 years	1.39 (0.84–2.31)	0.2000	1.46 (0.88–2.42)	0.1380
	40–59 years	2.27 (1.39–3.7)	0.0010*	2.47 (1.52–4.01)	0.0003*
	60–79 years	3.94 (2.42–6.41)	< 0.0001*	4.35 (2.68–7.07)	< 0.0001*
	80–100 years	6.7 (4.1–10.95)	< 0.0001*	8.01 (4.92–13.04)	< 0.0001*
Ann Arbor stage	Stage I (reference)				
	Stage II	1.44 (1.29–1.6)	< 0.0001*	1.48 (1.33–1.65)	< 0.0001*
	Stage III	1.76 (1.57–1.96)	< 0.0001*	1.81 (1.62–2.02)	< 0.0001*
	Stage IV	2.42 (2.19–2.68)	< 0.0001*	2.56 (2.32–2.83)	< 0.0001*
B symptoms	No (reference)				
	Yes	1.32 (1.24–1.41)	< 0.0001*	1.33 (1.25–1.42)	< 0.0001*
Chemotherapy	With (reference)				
	Without	1.86 (1.64–2.11)	< 0.0001*	2.28 (2.02–2.57)	< 0.0001*
First primary tumor	No (reference)				
	Yes	0.88 (0.82–0.94)	0.0003*	0.84 (0.78–0.9)	< 0.0001*
Marital status	Divorce (reference)				
	Married	0.87 (0.78–0.96)	0.0074*	0.84 (0.76–0.94)	0.0014*
	Other	0.76 (0.64–0.91)	0.0023*	0.77 (0.64–0.91)	0.0033*
	Separated	0.96 (0.69–1.34)	0.8100	0.93 (0.68–1.28)	0.6712
	Single	0.89 (0.79–1.02)	0.0880	0.91 (0.8–1.03)	0.1367
	Widowed	0.96 (0.84–1.09)	0.5000	0.96 (0.85–1.09)	0.5505
Median household income	< $50,000 (reference)				
	> $75,000	0.81 (0.73–0.89)	< 0.0001*	0.79 (0.72–0.87)	< 0.0001*
	$50,000–$74,999	0.91 (0.83–0.99)	0.0310*	0.89 (0.82–0.97)	0.0091*
Race	Asian (reference)				
	Black	0.94 (0.81–1.09)	0.4300	0.98 (0.84–1.14)	0.7941
	Other	0.55 (0.37–0.83)	0.0041*	0.53 (0.36–0.8)	0.0024*
	White	0.81 (0.73–0.9)	0.0001*	0.83 (0.74–0.92)	0.0003*
Radiation	With (reference)				
	Without	1.18 (1.09–1.28)	0.0001*	1.2 (1.1–1.3)	< 0.0001*
Site	Extranodal (reference)				
	Nodal	1.13 (1.05–1.22)	0.0011*	1.13 (1.05–1.21)	0.0010*
Surgery	With (reference)				
	Without	1.36 (1.18–1.56)	< 0.0001*	1.45 (1.27–1.66)	< 0.0001*
The sequence of systemic therapy and surgery	No systemic therapy and/or surgery (reference)				
	Other	2.18 (1.06–4.47)	0.0340*	2.2 (1.09–4.43)	0.0277*
	Systemic therapy after surgery	1 (0.87–1.15)	0.9700	1.03 (0.9–1.18)	0.6474
	Systemic therapy before surgery	1.4 (0.9–2.17)	0.1400	1.69 (1.12–2.54)	0.0124*
	Systemic therapy both before and after surgery	1.28 (0.72–2.27)	0.4000	1.26 (0.69–2.3)	0.4430
Treatment timing	$$\le$$ 1 month (reference)				
	> 1 month	0.79 (0.74–0.84)	< 0.0001*	0.78 (0.73–0.83)	< 0.0001*

*P < 0.05

### Model development and validation

**Fig. 2:**
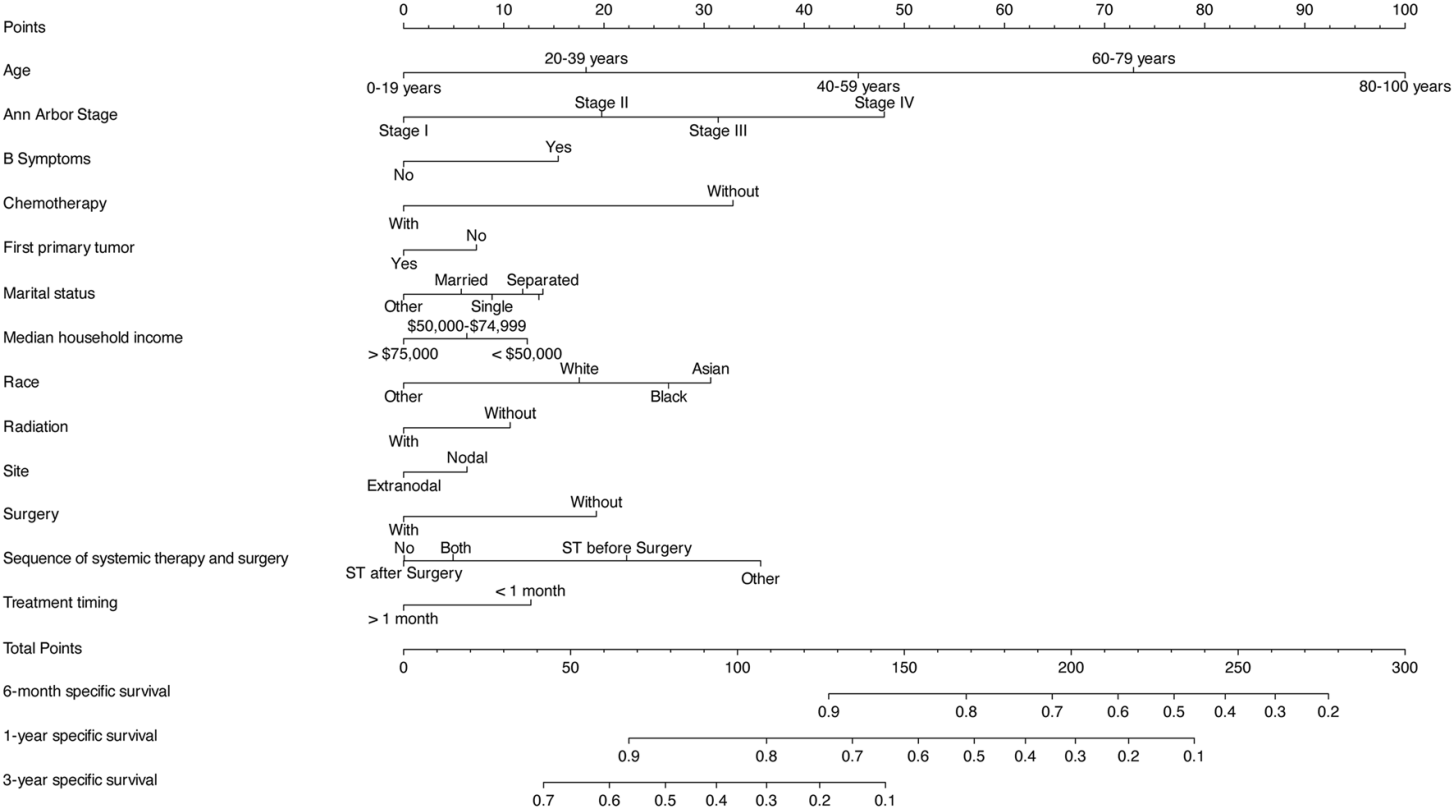
The nomogram of the competing risk model

**Fig. 3:**
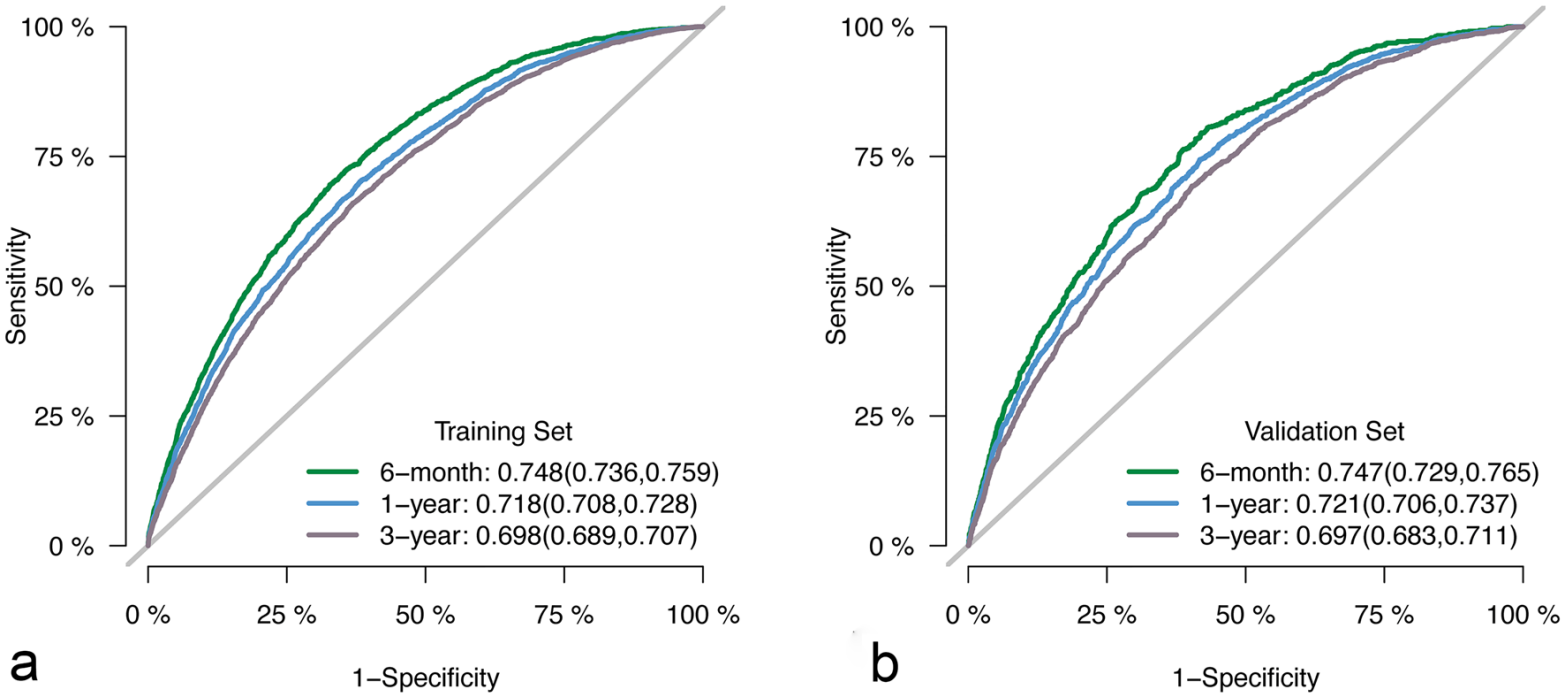
**a** The results of ROC curve analysis in the training cohort. **b** The results of ROC curve analysis in the validation cohort

**Fig. 4:**
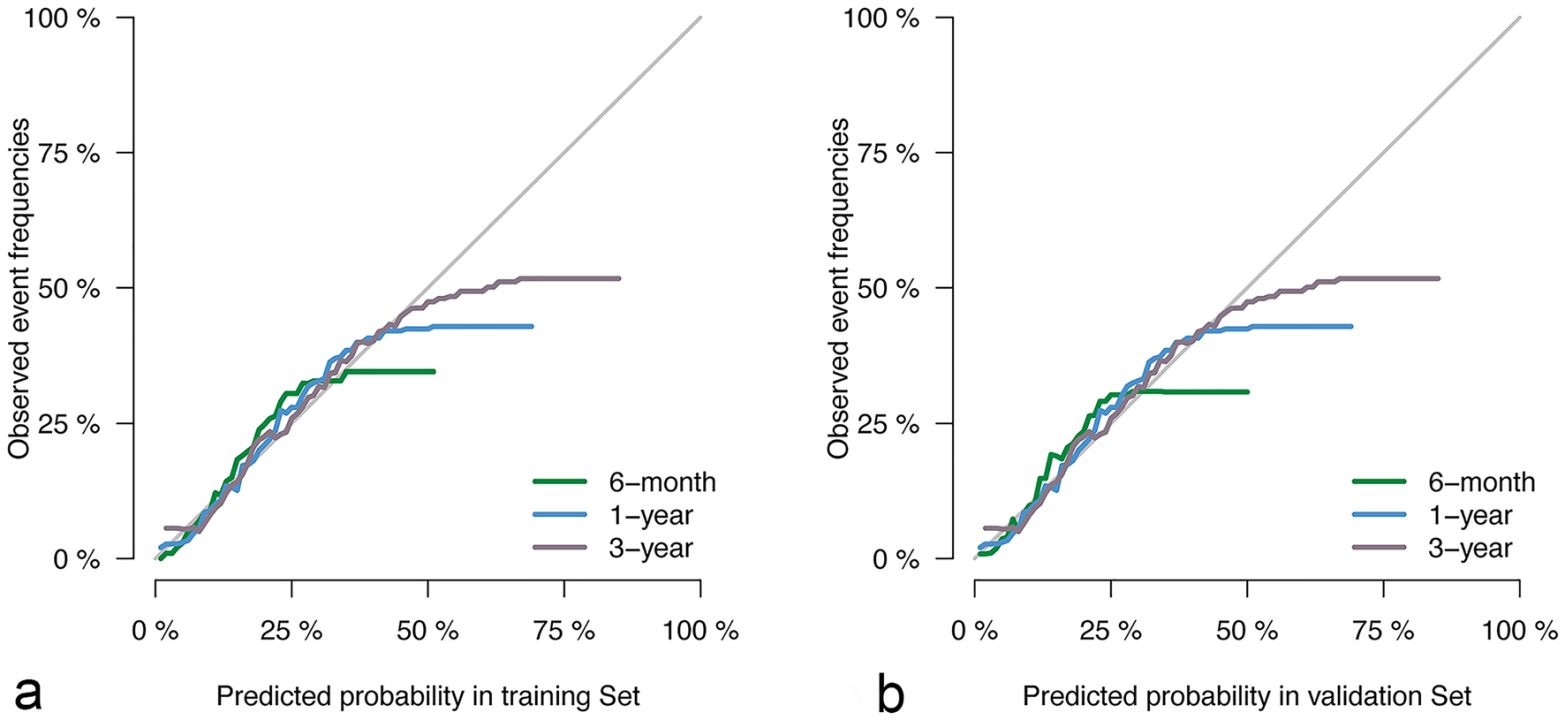
**a** The results of calibration curve analysis in the training cohort. **b** The results of calibration curve analysis in the validation cohort

The competing risk model incorporated 13 independent risk factors, achieving a C-statistic of 0.709 (± 0.002). To facilitate the application of this model, a corresponding nomogram, as shown in Fig. 2, was constructed. The points assigned to each individual variable were determined based on the patients’ classification, and the sum of these points yielded the Total Points. By matching the Total Points with the corresponding predictor, the cause-specific survival probability of patients could be estimated. The performance of the model was further evaluated through ROC curve analysis. The area under the curve (AUC) for 6-month, 1-year, and 3-year mortality in DLBCL patients was 0.748 (95% confidence interval [CI] [0.736, 0.759]), 0.718 (95% CI [0.708, 0.728]), and 0.698 (95% CI [0.689, 0.707]) in the training cohort, respectively. In the validation cohort, the AUC values were 0.747 (95% CI [0.729, 0.765]), 0.721 (95% CI [0.706, 0.737]), and 0.697 (95% CI [0.683, 0.711]), as depicted in Fig. 3. Moreover, the calibration curve analysis demonstrated that the model’s predicted results aligned closely with the actual values, as illustrated in Fig. 4. This further confirms the reliability and accuracy of the model in predicting outcomes.

## Discussion

We have devised a competing risk model in this research to forecast cause-specific mortality among DLBCL patients, which is then represented by a graphical nomogram. The model demonstrated favorable predictive accuracy and can offer reliable prognostic insights. This, in turn, may enhance clinicians’ comprehension of DLBCL and facilitate the provision of targeted clinical assistance to individuals at high risk.

The results of the feature selection demonstrated that there are 13 variables that serve as independent predictors of cause-specific mortality in DLBCL patients. These variables include race, tumor site (extranodal or nodal), Ann Arbor stage, surgery, radiation therapy, chemotherapy, sequence of systemic therapy and surgery, treatment timing, B Symptoms, whether it was the first primary tumor, age, marital status, and median annual household income. According to the results obtained from the nomogram, patient age was identified as the most accurate predictor, followed by Ann Arbor stage and chemotherapy. With regard to treatment, our study revealed that the absence of surgery, radiation therapy, chemotherapy, and systemic therapy was associated with a poorer prognosis for patients. It is widely acknowledged that chemotherapy is the primary treatment for DLBCL, and its efficacy has been supported by numerous studies [10, 11]. Radiation therapy is often used in conjunction with chemotherapy and has been shown to improve clinical symptoms in relapsed or refractory DLBCL patients following chemotherapy [12]. For the majority of lymphoma patients, chemotherapeutic agents are deemed more effective, thus surgical treatment is generally not recommended [13]. In fact, one study has shown that surgical treatment for lymphoma does not improve patient prognosis [14]. Nevertheless, there are certain specific cases where surgical intervention is necessary. For example, patients with primary gastrointestinal lymphoma may present with intestinal obstruction or splenomegaly alongside symptoms of compression [15]. In terms of demographic information, our findings suggest that Asians have a significantly higher mortality rate among DLBCL patients as compared to whites, and divorced patients exhibit a higher mortality rate than married patients. Furthermore, age [16], Ann Arbor stage [17], and B symptoms [18] have all been identified as predictors for cause-specific mortality in DLBCL patients, which is consistent with our study’s findings.

We utilized both the Fine and Gray model and the Cox proportional risk model to evaluate the influence of various variables on the outcome. To determine the impact of each variable, we computed the hazard ratio (HR). Table 3 illustrates the disparities between the variables in the two models. Considering the independent risk factors, we observed that age, Ann Arbor Stage, presence of b symptoms, absence of chemotherapy, absence of radiation, absence of surgery, and the sequence of systemic therapy and surgery significantly affected the risk of all-cause mortality in comparison to DLBCL-specific mortality. Regarding the independent protective factors, we found that the presence of a first primary tumor, marital status, median household income, and timing of treatment exerted a more pronounced influence on the risk of all-cause mortality compared to the risk of DLBCL-specific mortality. However, the effect of race (White) on the risk of all-cause mortality was relatively smaller.

Several studies have been conducted to assess the prognosis of patients with DLBCL using the SEER database. One particular study focused on the risk of developing second primary malignancies in DLBCL patients and revealed that the oral cavity and pharynx were the most vulnerable regions for malignant tumor development [19]. Other studies, encompassing diverse populations with DLBCL, investigated the prognosis of patients [20–23]. However, it is worth noting that the majority of these studies relied on the conventional Cox proportional risk model. In contrast, our study adopts a competing risk model, which takes into account both DLBCL-specific mortality events and the influence of competing events on the analysis outcomes. Most prognostic studies commonly utilize the traditional Kaplan–Meier method and Cox regression model to analyze survival patterns and identify significant prognostic indicators [24]. Nevertheless, real-world medical studies often involve the occurrence of multiple competing outcome events rather than a single event. Consequently, it becomes imperative to employ a competing risk model to mitigate the bias resulting from the presence of these competing risk events [25, 26]. The Competitive Risk Model, also known as the Fine and Gray model, was proposed by Fine and Gray in 1999 to address proportional risk situations in which competing risks are present. Unlike traditional survival models, this model focuses on modeling the subdistribution hazard function instead of the risk function for survival time. The subdistribution hazard function calculates the conditional risk of a specific event occurring before a certain point in time, considering the occurrence of competing events. This model is particularly useful when the endpoint event of a study, such as disease recurrence, can be “competed for” by other types of events, such as patient death from other causes. In such cases, traditional survival analysis methods may not provide accurate results. Using competing risk models, researchers can obtain more precise risk estimates and evaluate and compare the risk of specific events while accounting for the influence of other risk events.

The nomogram, a visual representation of models [27], has been widely recognized for its ability to depict complex relationships. Numerous studies have shown that machine learning models, such as random forests, neural networks, and support vector machines, can effectively capture nonlinear patterns in the data, thereby enhancing their predictive power [28–31]. However, one drawback of these models is their “black box” nature, which limits our understanding of the underlying computational process and the importance of each feature. In contrast, the nomogram offers a simple and intuitive graphical interface that allows for the quantification of the risk associated with each feature, making it particularly valuable for clinical applications [32]. The SEER database, maintained by the National Cancer Institute (NCI) [33], is a comprehensive and diverse collection of cancer incidence and survival data for specific populations in the United States. It serves as a valuable resource for researchers and healthcare professionals in understanding and analyzing cancer trends. With its large sample size and inclusion of multiple centers and racial backgrounds, the SEER database ensures that statistical findings derived from it are generally representative and reliable. This database offers detailed information on various aspects of cancer cases, such as patient demographics, cancer type, onset time, treatment approaches, and follow-up outcomes. Researchers can utilize this wealth of data to gain insights into the impact of cancer and develop effective strategies for diagnosis, treatment, and prevention.

In summary, an extensive dataset was utilized to develop a competing risk model for the prediction of cause-specific mortality in DLBCL patients. The model was effectively visualized as a nomogram and displayed favorable predictive performance, offering valuable information. However, it is crucial to acknowledge certain limitations within this study. Firstly, although the model exhibited satisfactory performance within both the training and validation cohorts, external validation remains necessary and is planned for the subsequent phase of our research. Secondly, due to constraints imposed by public databases, certain variables of interest were regrettably excluded from this investigation, including the specific chemotherapy agents administered to the patients. Furthermore, the lack of clarity in the categorization of certain variables within the database hinders the interpretation of their clinical significance. One such instance is the subcategory labeled as “Other”. Additionally, the potential impact of small subcategorical sample sizes on the model’s performance should be taken into consideration. However, it should be noted that the large sample sizes in this study mitigated this concern.

## Conclusion

Based on the SEER database, we have successfully developed a competing risk model for predicting the specific prognosis of DLBCL patients. The model has shown excellent performance in terms of its predictive accuracy. Among the various predictors evaluated, patient age emerges as the most crucial independent factor associated with DLBCL-specific mortality. Moreover, Ann Arbor stage and chemotherapy also demonstrate significant importance in predicting the prognosis. The clinical implications of our model are noteworthy as it aids clinicians in promptly identifying high-risk DLBCL patients. Consequently, this would facilitate the implementation of targeted clinical interventions and ultimately lead to improved patient outcomes.

### Supplementary Information

**Additional file 1: Table S1.** The results of univariate and multivariate analysis in competing risk model.

## Data Availability

On reasonable request, the corresponding author will provide the information supporting the study’s conclusions.
