# Supplementary material for: Specific mortality in patients with diffuse large B-cell lymphoma: a retrospective analysis based on the surveillance, epidemiology, and end results database
Source: Eur J Med Res. 2024 Apr 20;29:241. doi: 10.1186/s40001-024-01833-4 (PMC11031870; doi:10.1186/s40001-024-01833-4)
Supplement: Supplementary file 1 — Additional file 1: Table S1. The results of univariate and multivariate analysis in competing risk model. [file 40001_2024_1833_MOESM1_ESM.docx]

**Additional file 1**

**Table S1** The results of univariate and multivariate analysis in competing risk model.

| Factors | Levels | Univariate analysis | | Multivariate analysis | |
| --- | --- | --- | --- | --- | --- |
|  |  | HR^1^ (95% CI^1^) | P value | HR (95% CI) | P value |
| Age | 0-20 years (Reference) |  |  |  |  |
|  | 20-39 years | 1.37(0.83-2.25) | 0.2200 | 1.38(0.83-2.29) | 0.2100 |
|  | 40-59 years | 2.33(1.44-3.76) | 0.0006* | 2.24(1.37-3.65) | 0.0012* |
|  | 60-79 years | 3.98(2.47-6.41) | < 0.0001* | 3.88(2.38-6.32) | < 0.0001* |
|  | 80-100 years | 6.81(4.21-10.99) | < 0.0001* | 6.64(4.06-10.84) | < 0.0001* |
| Ann Arbor Stage | Stage I (Reference) |  |  |  |  |
|  | Stage II | 1.42(1.28-1.58) | < 0.0001* | 1.37(1.22-1.53) | < 0.0001* |
|  | Stage III | 1.96(1.77-2.16) | < 0.0001* | 1.64(1.45-1.84) | < 0.0001* |
|  | Stage IV | 2.61(2.39-2.86) | < 0.0001* | 2.3(2.07-2.56) | < 0.0001* |
| B Symptoms | No (Reference) |  |  |  |  |
|  | Yes | 1.4(1.32-1.48) | < 0.0001* | 1.31(1.23-1.4) | < 0.0001* |
| Chemotherapy | With (Reference) |  |  |  |  |
|  | Without | 1.69(1.53-1.87) | < 0.0001* | 1.88(1.66-2.13) | < 0.0001* |
| First primary tumor | No (Reference) |  |  |  |  |
|  | Yes | 0.72(0.67-0.77) | < 0.0001* | 0.87(0.81-0.94) | 0.0002* |
| Marital status | Divorce (Reference) |  |  |  |  |
|  | Married | 0.87(0.78-0.96) | 0.0064* | 0.87(0.78-0.96) | 0.0083* |
|  | Other | 0.7(0.59-0.83) | 0.0001* | 0.77(0.64-0.92) | 0.0034* |
|  | Separated | 0.88(0.64-1.21) | 0.4200 | 0.98(0.7-1.36) | 0.8800 |
|  | Single | 0.66(0.59-0.75) | < 0.0001* | 0.9(0.79-1.02) | 0.1100 |
|  | Widowed | 1.35(1.19-1.52) | < 0.0001* | 0.96(0.85-1.09) | 0.5500 |
| Median household income | < $50,000 (Reference) |  |  |  |  |
|  | > $75,000 | 0.83(0.75-0.91) | 0.0001* | 0.81(0.73-0.89) | < 0.0001* |
|  | $50,000-$74,999 | 0.92(0.85-1) | 0.0580 | 0.91(0.83-0.99) | 0.0240* |
| Number of malignant tumors | > 1 (Reference) |  |  |  |  |
|  | 1 | 1(0.94-1.06) | 0.9400 |  |  |
| Place of residence | Metropolitan (Reference) |  |  |  |  |
|  | Nonmetropolitan | 1.05(0.96-1.15) | 0.2800 |  |  |
| Primary site | Digestive system (Reference) |  |  |  |  |
|  | Intra-abdominal lymph nodes | 1.33(1.1-1.62) | 0.0040* | 1.17(0.94-1.46) | 0.1700 |
|  | Intrathoracic lymph nodes | 0.86(0.66-1.12) | 0.2600 | 1.03(0.77-1.36) | 0.8600 |
|  | Lymph nodes of axilla or arm | 0.76(0.57-1.03) | 0.0740 | 0.78(0.57-1.07) | 0.1300 |
|  | Lymph nodes of head, face & neck | 0.7(0.57-0.87) | 0.0014* | 0.8(0.63-1.02) | 0.0700 |
|  | Lymph nodes of inguinal region or leg | 0.89(0.68-1.17) | 0.4100 | 0.92(0.68-1.24) | 0.5800 |
|  | Lymph nodes of multiple regions | 1.43(1.21-1.69) | < 0.0001* | 1.1(0.9-1.34) | 0.3500 |
|  | Nervous system | 1.11(0.16-7.6) | 0.9200 | 1.34(0.13-13.89) | 0.8000 |
|  | Other | 1.1(0.93-1.3) | 0.2700 | 1.01(0.84-1.21) | 0.9200 |
|  | Pelvic lymph nodes | 1.45(1.05-2.02) | 0.0260* | 1.27(0.9-1.8) | 0.1700 |
| Race | Asian (Reference) |  |  |  |  |
|  | Black | 0.91(0.79-1.05) | 0.1900 | 0.94(0.81-1.09) | 0.4100 |
|  | Other | 0.58(0.39-0.87) | 0.0081* | 0.54(0.36-0.82) | 0.0033* |
|  | White | 0.93(0.84-1.03) | 0.1500 | 0.81(0.73-0.89) | < 0.0001* |
| Radiation | With (Reference) |  |  |  |  |
|  | Without | 1.4(1.3-1.52) | < 0.0001* | 1.16(1.07-1.26) | 0.0003* |
| Sex | Female (Reference) |  |  |  |  |
|  | Male | 1.01(0.95-1.07) | 0.7300 |  |  |
| Site | Extranodal (Reference) |  |  |  |  |
|  | Nodal | 1.33(1.24-1.43) | < 0.0001* | 1.11(1-1.22) | 0.0400* |
| Surgery | With (Reference) |  |  |  |  |
|  | Without | 1.33(1.23-1.43) | < 0.0001* | 1.35(1.17-1.55) | < 0.0001* |
| The sequence of systemic therapy and surgery | No systemic therapy and/or surgery (Reference) |  |  |  |  |
|  | Other | 1.79(0.88-3.65) | 0.1100 | 2.21(1.08-4.55) | 0.0300* |
|  | Systemic therapy after surgery | 0.71(0.65-0.77) | < 0.0001* | 1(0.87-1.15) | 0.9500 |
|  | Systemic therapy before surgery | 1.02(0.67-1.54) | 0.9300 | 1.41(0.91-2.19) | 0.1300 |
|  | Systemic therapy both before and after surgery | 1.01(0.57-1.79) | 0.9800 | 1.26(0.71-2.22) | 0.4300 |
| Treatment timing | < 1 month (Reference) |  |  |  |  |
|  | > 1 month | 0.88(0.83-0.93) | < 0.0001* | 0.79(0.74-0.84) | < 0.0001* |

^1^: HR: hazard ratio; CI: confidence interval; *: P < 0.05.
